# The Genetic Architecture of Seed Composition in Soybean Is Refined by Genome-Wide Association Scans Across Multiple Populations

**DOI:** 10.1534/g3.114.013433

**Published:** 2014-09-22

**Authors:** Justin N. Vaughn, Randall L. Nelson, Qijian Song, Perry B. Cregan, Zenglu Li

**Affiliations:** * Center for Applied Genetic Technologies and Department of Crop and Soil Sciences, University of Georgia, Athens, Georgia 30602; † Soybean Maize Germplasm, Pathology, and Genetics Research Unit, USDA, Agricultural Research Service, and Department of Crop Sciences University of Illinois, Urbana, Illinois 61801; ‡ Soybean Genomics and Improvement Laboratory, USDA, Agricultural Research Service, Beltsville, Maryland 20705

**Keywords:** genome-wide association, QTL, protein, oil, amino acid, soybean population structure

## Abstract

Soybean oil and meal are major contributors to world-wide food production. Consequently, the genetic basis for soybean seed composition has been intensely studied using family-based mapping. Population-based mapping approaches, in the form of genome-wide association (GWA) scans, have been able to resolve loci controlling moderately complex quantitative traits (QTL) in numerous crop species. Yet, it is still unclear how soybean’s unique population history will affect GWA scans. Using one of the populations in this study, we simulated phenotypes resulting from a range of genetic architectures. We found that with a heritability of 0.5, ∼100% and ∼33% of the 4 and 20 simulated QTL can be recovered, respectively, with a false-positive rate of less than ∼6×10^−5^ per marker tested. Additionally, we demonstrated that combining information from multi-locus mixed models and compressed linear-mixed models improves QTL identification and interpretation. We applied these insights to exploring seed composition in soybean, refining the linkage group I (chromosome 20) protein QTL and identifying additional oil QTL that may allow some decoupling of highly correlated oil and protein phenotypes. Because the value of protein meal is closely related to its essential amino acid profile, we attempted to identify QTL underlying methionine, threonine, cysteine, and lysine content. Multiple QTL were found that have not been observed in family-based mapping studies, and each trait exhibited associations across multiple populations. Chromosomes 1 and 8 contain strong candidate alleles for essential amino acid increases. Overall, we present these and additional data that will be useful in determining breeding strategies for the continued improvement of soybean’s nutrient portfolio.

Soybeans are a primary contributor to the worldwide production of culinary oils and animal feed. They also serve as a significant source of renewable biofuel, in the form of biodiesel (Hill *et al.* 2006). Given the importance of protein and oil content to soybean producers, the quantitative trait loci (QTL) underlying these traits have undergone intensive investigation (Diers *et al.* 1992; Chung *et al.* 2003; Fasoula *et al.* 2004; Nichols *et al.* 2006; Bolon *et al.* 2010; Hwang *et al.* 2014). Although many QTL have been identified across the soybean genome, a region of linkage group (LG) I has consistently shown the strongest association with percent protein composition of the seed (Diers *et al.* 1992; Chung *et al.* 2003; Nichols *et al.* 2006). A high-protein allele on LG-I has been introgressed from soybean’s wild progenitor, *Glycine soja* (Diers *et al.* 1992; Sebolt *et al.* 2000); therefore, this allele is likely to be dispersed throughout global soybean germplasm. This assumption is supported by the identification of the LG-I region from mapping populations derived by crossing high-/low-protein *Glycine max* lines (Wilcox and Cavins 1995; Chung *et al.* 2003; Fasoula *et al.* 2004). Although breeders would like to increase both protein and oil content, the traits exhibit a strong negative correlation that has not been possible to decouple (Wilson 2004). Moreover, total yield is often negatively correlated with percent protein, although the correlation is weaker than protein by oil (Chung *et al.* 2003). It is possible that the two traits are controlled by the same causal variant, or that separate causal variants are in very tight linkage—previously calculated as <0.67 cM (Chung *et al.* 2003). Because many legumes do not exhibit such sharp correlations in protein and oil (Sarvamangala *et al.* 2011), there is hope that in soybean the traits can be separated via recombination of the underlying genes. Alternatively, if the traits are determined by a single variant, it remains of great commercial and biochemical interest how this gene might be mediating such large-scale effects.

Protein meal is a major source of feed for poultry and swine, which are unable to synthesize the entire suite of required amino acids and therefore must derive these nutrients from their diets. It follows that the value of soybean meal is less a function of its crude protein content and more a function of its amino acid profile (Friedman and Brandon 2001). While the manipulation of other aspects of seed composition and processing may improve amino-acid assimilation, increasing the relative proportion of methionine (Met), lysine (Lys), and threonine (Thr) has become a goal in soybean breeding (Durham 2003). The economic benefit of improved essential amino acid content has been estimated to be ∼$5 per ton per 10% increase of any of the above amino acids (Clarke and Wiseman 2000).

Unlike the total protein trait, few studies have attempted to map protein quality QTL. Generally, there is only a weak correlation between crude protein content and Met and Cysteine (Cys) levels (Burton *et al.* 1982; Wilcox and Shibles 2001). Sulfur-containing amino acid composition does tend to fluctuate depending on the nitrogen source (Paek *et al.* 1997) and availability of reduced forms of sulfur (Grabau *et al.* 1986), thus environmental effects are likely to play a significant role in phenotypic outcomes. In mapping studies, 100 markers were used to genotype ∼100 F_6_ recombinant inbred lines (RILs) (Panthee *et al.* 2006a,b). Nearly 10% of RILs had a Met+Cys value exceeding the United Nations’ Food and Agriculture Organization standards based on egg protein (Clarke and Wiseman 2000). Moreover, Met concentrations in some RILs were 30% higher than the parent values, which were approximately equivalent, indicating that both parent lines contributed positive alleles (Panthee *et al.* 2006a). The heritability of these traits appears to be moderate for Met (0.56) and low for Cys (0.14), and they are effected by maturity date when grown in the same environment (Panthee *et al.* 2006a).

As described above, nearly all work on mapping seed composition QTL has focused on family-based populations, either as selfed populations or near-isogenic lines. Because of the limited amount of recombination that has occurred in these populations, such studies generally have a limited genetic resolution of the QTL. To improve resolution, linkage disequilibrium (LD) mapping or population-based association mapping uses a diverse set of plant accessions, which have a much lower LD than a family-based population (Myles *et al.* 2009). This reduced LD requires much higher marker density than family-based mapping. Also, population-based mapping is confounded when sites with divergent allele frequencies across subpopulations of the panel are mistaken for causal sites because the polygenic background effect dominates the signal of any one true site (Vilhjálmsson and Nordborg 2013). Generally, these false positives can be removed by controlling for relatedness among individuals, which are a proxy and predictor of the sum of un-modeled genetic effects (Yu *et al.* 2006; Segura *et al.* 2012). Many methods have been developed to account for these biases, and genome-wide association (GWA) scans have identified known genes in numerous crops and model organisms, including maize (Buckler *et al.* 2009), rice (Atwell *et al.* 2010; Huang *et al.* 2010), Arabidopsis (Atwell *et al.* 2010), and foxtail millet (Jia *et al.* 2013). Although this collection of species has a broad range of mating systems and population histories, soybean represents an extreme case of inbreeding (Hyten *et al.* 2006; Chung *et al.* 2013). In addition, it has undergone one or more substantial population bottlenecks during domestication (Hyten *et al.* 2006) and, as with other crops, numerous loci have been under strong artificial selection (Chung *et al.* 2013). The performance of available GWA methods has yet to be rigorously investigated in soybean.

In the following study, we aim to explore the utility of GWA scans in soybean, to refine QTL for assorted seed composition traits, and to assess the relationship between population structure and genetic architecture with regard to these traits.

## Materials and Methods

### Phenotype and genotype data

Phenotypic data for protein and oil were supplied by the Germplasm Resources Information Network (http://www.ars-grin.gov/cgi-bin/npgs/html/crop.pl?51) and are designated as IL-1964, IL-1966, MS-1996, and MS-2000 (Supporting Information, Table S1). IL-1964 included data from 619 accessions in maturity groups (MGs) I and II that were introduced into the United States before 1961, as well as U.S. varieties released prior to 1965. They were evaluated in Urbana, Illinois, in two replications in 1964 (Bernard *et al.* 1998). The first replication was planted on May 14 and the second replication was planted on June 3. IL-1966 had data from 977 introduced soybeans and U.S.-released cultivars in MGs III and IV from the same time period as IL-1964. These lines were evaluated in Urbana, Illinois, in 1965 and 1966, with one replication each year (Bernard *et al.* 1998). MS-1996 included 728 accessions in MGs V through IX. These were accessions introduced into the United States or were released as cultivars generally between 1990 and 1994; however, some cultivars and introduced accessions that predated this time were also included. They were evaluated at Stoneville, Mississippi, with one replication in 1996 and a second replication in 1997 (Peregrine *et al.* 2008). MS-2000 had data from 934 accessions in MGs IV through VI, although almost all were in MG V. They were accessions introduced into the United States prior to 1977 as well as selected U.S. cultivars released between 1980 and 1991. These entries were evaluated at Stoneville, Mississippi, in 1999 and 2001, with one replication grown in each year (Peregrine *et al.* 2008). Oil and protein concentrations used in GWA scans were the average of replicates for a genotype.

For amino acid and sugar composition, lines were grown in Illinois in 1996 (IL-1996) and in Mississippi in 1997 (MS-1997). Single soybean samples were analyzed by NIR at the University of Minnesota’s Soybean Breeding Laboratory. Whole soybean samples received from the USDA Soybean Germplasm Collection were ground and then analyzed on a FOSS 6500 NIR Instrument. NIR Spectra from the FOSS 6500 were predicted using ISIPredict Software version 1.10.2.4842. Calibrations, provided by FOSS North America, were used to predict soybean composition from the NIR spectra.

The populations used for the protein and oil GWA scans (Table S1) do not overlap with one another in terms of genotypes. For example, a genotype in the MS-2000 population will not also appear in the IL-1966 population. Similarly, for amino acid GWA scans (Table S1), genotypes will not overlap between populations. All phenotypic data are provided (File S1).

Genotypic data were derived from the large-scale effort to genotype the USDA Soybean Germplasm Collection using SoySNP50K iSelect BeadChips (Song *et al.* 2013). The data were accessed from http://soybase.org/data_distribution/soybase_soy50K_snp_all_cultivars_and_snps.gz on 6 February 2014. Missing data in the total genotyped data set were imputed using command-line TASSEL 3.0 and default *-impute* options (Bradbury *et al.* 2007). To reduce computation demands, imputation was performed using 1000 genotypes at a time. For a given trait and population, genotypes were filtered from the total data set and markers with a minor allele frequency of <0.05 were removed. Generally, filtering resulted in ∼32,000 SNP markers per population, although the number of SNP markers for the MS-2000 population was 28,622 (Table S1). Physical distances described in this manuscript are based on genome assembly version Glyma.Wm82.a1 (Gmax1.01); the distances are shifted in version Glyma.Wm82.a2 (Gmax2.0) (Schmutz *et al.* 2010).

### Kinship

Kinship among lines was calculated in the manner of Vanraden (2008) using an R implementation (www.R-project.org) available as part of GAPIT software libraries (Lipka *et al.* 2012). Using resultant distances, clustering was performed in R using the internal package *hclust* with default parameters.

### Simulations

QTL were randomly selected from all available markers within the MS-2000 population (Figure 2A). Only markers with MAF >0.05 were used. Two types of effect distributions were used: uniform, for which each QTL has an equal effect (1/*n*; where *n* = total QTL), and linear, for which QTL effects start at [1/(*n* – ((*n* – 1)!/*n*))] and decline in a linear fashion toward 0, such that the nonzero effects sum to 1, regardless of the number of loci. In other words, 1 is the maximum total genotypic effect in any simulation. Phenotypes were simulated by adding residual effects that were drawn from a normal distribution with a mean of 0 and dispersion dependent on the defined heritability (Wimmer *et al.* 2013). Five replicates per condition (Table 1) were run as described below.

**Table 1:** Simulation results using MS-2000 population

#QTL	4				20				200	
Effect Distr.	Linear		Uniform		Linear		Uniform		Linear	
H^2^	0.95	0.5	0.95	0.5	0.95	0.5	0.95	0.5	0.95	0.5
False (−)*^d^*	0.1*^b^* (0.1*^c^*)	0.35 (0.25)	0 (0)	0.1 (0)	0.5 (0.41)	0.69 (0.63)	0.3 (0.19)	0.68 (0.53)	0.96 (0.93)	0.99 (0.99)
(total)										
False (−)	0 (0)	0 (0)	NA*^a^*	NA	0.04 (0.04)	0.24 (0.12)	NA	NA	0.87 (0.78)	0.98 (0.97)
(top 1/4)*^a^*										
False (−)	0 (0)	0.13 (0.13)	NA	NA	0.33 (0.23)	0.59 (0.51)	NA	NA	0.94 (0.91)	0.99 (0.99)
(top 3/4)*^a^*										
False (+)	2.0E−4 (3.5E−4)	4.2E−5 (4.2E−5)	3.5E−5 (4.9E−5)	4.9E−5 (6.3E−5)	3.6E−4 (8.5E−4)	8.4E−5 (1.0E−4)	2.9E−3 (4.8E−3)	8.4E−5 (2.0E−4)	2.8E−5 (1.9E−4)	1.4E−5 (4.2E−5)

Each value is the mean of five separate replicates under the given combination of variables.

a “Top 1/4” indicates that only the top quartile of loci with the strongest effects were evaluated in terms of type II errors. Similarly, “Top 3/4” refers to the top 3 quartiles. Because these categories cannot apply to uniform effect distributions, applicable cells are given “NA” values.

b *p*-value threshold < 10^−5^.

c Parenthetical values for *p*-value threshold <10^−4^.

d False (−) indicates the fraction of true positives that were missed; false (+) indicates the fraction of tests that identified untrue associations.

### GWA scans

For compressed mixed linear model (CMLM) analysis (Zhang *et al.* 2010), GAPIT (Lipka *et al.* 2012) was used with group size increments set to 20 (group.by = 20), the number of principal components (PCs) used set to 3 (PCA.total = 3), and the number of markers sampled to estimate kinship was set to 80% (SNP.fraction = 0.8). Three PCs were used because improvements in fit generally diminish after three PCs, and because the third PC differentiates Japanese from South Korean germplasm (Figure S4, Figure S5, Figure S6, Figure S7). For the multi-locus mixed model (MLMM) analysis (Segura *et al.* 2012), we used the Python implementation (https://github.com/bvilhjal/mixmogam, version 1.0) with minor modifications to support our data formats. Because the inclusion of heterozygous genotypes dramatically reduces the number of possible markers used in this implementation, heterozygotes were randomly assigned to be homozygous for an allele based on total allele frequencies at that SNP marker. This was only with regard to implementing the MLMM analysis; also, because heterozygous loci are rare in the majority of soybean lines, only ∼1 in every 1000 SNPs was actually altered, adding minor, nonsystematic noise to the MLMM analysis. Only markers with a significant association (*p*-value <10^−4^) under both CMLM and MLMM were reported (Table 2 and Table 3). Heritability, or pseudo-heritability, estimates are generated by the EMMA algorithm (Kang *et al.* 2008) that is used to control for population structure in the MLMM (Table 2 and Table 3). Allelic effects are estimated in the CMLM model, with the top three PCs included and population structure accounted for (Table 2 and Table 3).

**Table 2:** GWA scan results for protein and oil

MS-1996 (728)*^a^*			MS-2000 (934)			IL-1964 (619)			IL-1966 (977)			Huang *et al.* 2014 (214)		
SNP	-log (*p*-value)*^b^*	Allelic Effect Estimate	SNP	-log (*p*-value)	Allelic Effect Estimate	SNP	-log (*p*-value)	Allelic Effect Estimate	SNP	-log (*p*-value)	Allelic Effect Estimate	SNP	-log (*p*-value)	Allelic Effect Estimate
% Protein (43.45 [43.50])*^c^* ; H^2^ = 0.65 (MS-1966), 0.80 (MS-2000), 0.52 (IL-1964), 0.79 (IL-1966), 0.88 (Hwang 2014)														
***12_38378352****^d^*,*^e^*	4.85 (4.69)	0.6	***20_31972955***	10.63 (10.43)	1.38	8_44632488	4.4 (4.37)	0.44	13_24858209	4.75 (4.12)	0.39	***20_31610452***	5.99 (5.98)	1.49
17_2678979	4.03 (4.61)	0.63	***12_34729290***	4.94 (4.11)	0.66				11_37932701	4.68 (5.08)	0.41			
									15_3919945	4.35 (4.24)	0.28			

% Oil (18.60 [18.45]); H^2^ = 0.66 (MS-1966), 0.88 (MS-2000), 0.73 (IL-1964), 0.78 (IL-1966), 0.91 (Hwang 2014)														
15_12490330	6.43 (6.9)	0.34	***20_31150279***	13.33 (15.02)	1.06	20_613396	6.51 (6.56)	0.46	12_39580013	6.32 (5.6)	0.22	***20_31150279***	5.27 (5.2)	0.71
**6_46040638**	4.72 (5.32)	0.28	**15_3919945**	8.15 (8.04)	0.48	***15_3702534***	7.83 (5.8)	0.72	6_10324739	4.67 (4.5)	0.28	16_7253088	4.54 (4.64)	0.62
7_35214966	5.11 (4.6)	0.57	**5_38495217**	6.74 (8.39)	0.78	11_11633102	4.56 (5.78)	0.61	4_2052748	4.51 (4.61)	0.33			
**5_38495217**	4.88 (4.6)	0.28	**6_42907701**	5.7 (4.31)	0.26	14_10300717	4.49 (4.33)	0.36						
16_510565	4.64 (4.81)	0.44				8_2969485	4.45 (5.1)	0.5						
						15_3828587	4.49 (4.33)	0.36						
						4_9617727	4.22 (4.42)	0.51						

a Population used; value in parentheses is the number of genotypes used.

b Value from MLMM; parenthetical value from CMLM.

c Mean values for combined populations are in parentheses with median values in brackets.

d Bold font indicates that the marker (or a marker within 4 Mbp) was associated with the trait in two or more environment–population datasets.

e Italic font indicates that the marker (or a marker within 4 Mbp) was also associated with another trait in the study.

**Table 3:** GWA scan results for selected essential amino-acid profiles

IL-1996 (900)*^a^*			MS-1997 (978)		
SNP	-log (*p*-value)*^b^*	Allelic Effect Estimate	SNP	-log (*p*-value)*^b^*	Allelic Effect Estimate
Cysteine (1.47 [1.50])*^c^* ; H^2^ = 0.60 (IL-1996), 0.59 (MS-1997)					
***8_8462762****^d^*,*^e^*,*^f^*	5.39 (5.01)	0.06	***8_8462762***	12.33 (12.06)	0.06
			6_18690983	9.87 (8.6)	0.04
			6_17674401	4.33 (5.01)	0.03

Lysine (6.41 [6.47]); H^2^ = 0.61 (IL-1996), 0.66 (MS-1997)					
***8_8462762***	11.1 (10.97)	0.28	***8_8577294***	11.01 (9.55)	0.11
17_39726391	6.96 (6.52)	0.14	12_14812823	8.87 (6.04)	0.1
*1_52249479*	6.16 (4.79)	0.08	9_41819055	6.25 (6.36)	0.14
16_28941919	4.46 (5.31)	0.13	17_270328	6.32 (5.46)	0.1
			15_23117810	5.29 (4.62)	0.07
			19_41918030	4.92 (4.72)	0.09

Methionine (1.43 [1.40]); H^2^ = 0.75 (IL-1996), 0.73 (MS-1997)					
11_1657825	11.47 (13.47)	0.08	***1_52263952***	7.46 (6.6)	0.03
20_694345	8.73 (8.86)	0.1	5_29970914	6.19 (5.28)	0.04
3_3936105	7.5 (6.78)	0.06	2_51258638	5.03 (4.51)	0.04
***1_52253980***	4.88 (5.26)	0.04	20_43025938	4.12 (6.42)	0.04
16_36220954	4.91 (4.88)	0.05	*8_8577294*	6.48 (4.03)	0.03
3_40763529	4.39 (5.26)	0.05	10_40692799	4.21 (4.42)	0.03

Threonine (3.57 [3.60]); H^2^ = 0.56 (IL-1996), 0.65 (MS-1997)					
*8_8627848*	6.93 (5.51)	0.08	*1_52253980*	10.71 (11.68)	0.06
			4_6897543	4.21 (4.17)	0.04

Sucrose (4.00 [3.83]); H^2^ = 0.66 (IL-1996), 0.50 (MS-1997)					
18_59597832	5.19 (4.67)	0.38	5_38495217	5.05 (4.99)	0.35
15_12181005	4.43 (5.05)	0.29			

Stachyose (2.89 [2.76]); H^2^ = 0.27 (IL-1996), 0.40 (MS-1997)					
9_2007514	5.21 (5.58)	0.17	20_2219331	4.05 (4.06)	0.14

Associated Manhattan plots are given in Figure S1 and Figure S2.

a Population used; value in parentheses is the number of genotypes used.

b Value from MLMM; parenthetical value from CMLM.

c Mean values for combined populations are in parentheses, with median values in brackets (% protein by dry weight).

d Bold font indicates that the marker (or a marker within 200 Kbp) was associated with the trait in two or more environment–population datasets.

e Italic font indicates that the marker (or a marker within 200 Kbp) was also associated with another trait in the study.

f For simplicity, marker names are reduced to their chromosome position form, *e.g.*, BARC1.01Gm08_8462762 appears as 8_8462762.

## Results and Discussion

### Population structure and protein/oil traits

**Figure 1:**
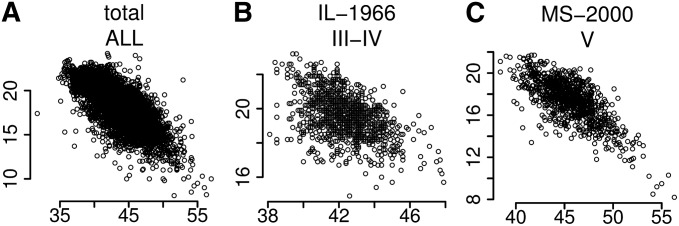
Protein and oil phenotypic variation and covariance within populations. (A) All lines across all populations for which protein and oil were measured. (B and C) Lines for a specific population assayed in a particular environment. IL, Illinois; MS, Mississippi. Year of growth is given adjacent to location and maturity group is given below the location and date. In all graphs, percent dry-weight protein and oil are plotted on the x-axis and y-axis, respectively.

Using phenotypic data accumulated through the USDA Germplasm Resources Information Network, we initially focused on the two major constituents of soybean seed—protein and oil. Phenotypic data are either the average of multi-year trials, as in the MS-2000 population, or the result of a single season trial, as in the IL-1966 (Table S1). As known from numerous previous studies, there is a strong negative correlation between protein and oil content. Across the entire data set, which represents a range of environments and maturity groups, protein data used in this study have a −0.65 correlation coefficient with oil (Figure 1A). Even when limiting the analysis to specific environments and maturity groups, the inverse relationship still exists (Figure 1, B and C). As noted previously, Southern lines (MG V, Figure 1B), as a group, have greater phenotypic variation toward the high end of protein content, whereas Northern germplasm is missing high-protein lines. Additionally, the correlation between protein and oil in Northern lines is approximately two-fold weaker than Southern lines, although it is still present (Figure 1C).

**Figure 2:**
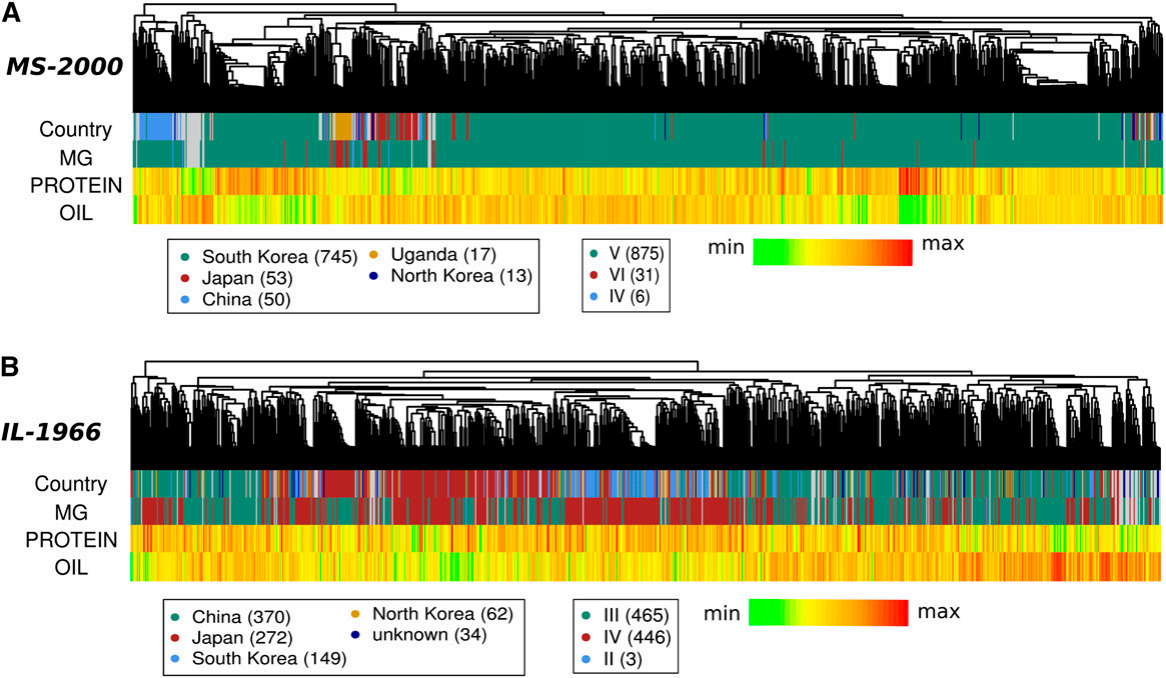
Attributes of populations used in GWA scans for protein and oil. Genotypes were clustered based on genetic distance. Each genotype used in the study represents a leaf in the dendrogram at the top of each panel. Country of origin and maturity group (“MG”) are color-coded. Protein and oil are represented as a heat map, with red being the highest value within that population and green being the lowest. Values in parentheses indicate the number of lines within a given category. (A) MS-2000 population. (B) IL-1966 population. Note that color-coding can be different for the same category in different populations.

We explored the genetic architecture of these traits further with regard to the relatedness structure within each population. All soybean accessions in the USDA Soybean Germplasm Collection have been genotyped with Soy50K SNP Infinium Chips (Song *et al.* 2013), which contain ∼50,000 markers based on population-wide single-nucleotide polymorphisms (SNPs). Extracting phenotyped lines from this dataset, we calculated kinship matrices for both MS-2000 and IL-1966 populations. Lines within each population were then clustered based on their identity by descent estimation (represented as dendrograms in Figure 2). The MS-2000 population primarily comprises germplasm with South Korean origins (Figure 2A). There is clear distinction between the South Korean germplasm and Chinese germplasm, whereas Japanese germplasm clusters more closely with the South Korean population (Figure 2, A and B, and Figure S5). There also appears to be a subpopulation of the South Korean germplasm that is quite distinct from both Japanese and other South Korean sources. Because populations comprise lines from similar maturity groups, we see very little clustering based on this factor. These results are characteristic of all populations analyzed and supported by principal component analysis (Figure S4, Figure S5, Figure S6, Figure S7).

The inverse relationship between protein and oil is reflected in Figure 2 (*e.g.*, if oil is red, then protein is green). Within both populations, very closely related accessions tend to share similar protein/oil levels, although the observation is less pronounced in the IL-1966 population, where less structure is present. Protein levels are generally not related to maturity group (MG in Figure 2). Thus, the traits appear to have a strong genetic basis. We further interrogated the genetic architecture of seed composition using GWA scans.

### Detection success of GWA studies in soybean populations

Although there have been GWA studies performed in soybean, these have generally relied on between 500 and 1500 SNP markers (Mamidi *et al.* 2011; Hao *et al.* 2012). Even though soybean populations generally have very high LD, such sparse marker density will often fail to identify significant associations in a panel because recombination has physically and statistically decoupled a causal variant from nearby markers. Although higher-density marker studies have improved this aspect of GWA scans (Hwang *et al.* 2014), these have relied on preexisting models and algorithms, namely the compressed mixed model (Yu *et al.* 2006; Zhang *et al.* 2010). To test the utility of preexisting GWA tools on actual soybean association panels, we simulated hypothetical phenotypes under a range of genetic architectures using the MS-2000 population of genotypes described above (Figure 2 and Table S1).

We generated five replicates for each combination of parameters defined in Table 1 (*e.g.*, for the combination with 20 QTL, a linear effect distribution, and a H^2^ of 0.5, five separate simulations were performed in which a new set of QTL positions were randomly selected). Generally, the heritability of protein and oil content is expected to lie within the simulated range (Chung *et al.* 2003). We used only additive models; heterozygous genotypes, though few, were calculated as exactly intermediate between homozygous genotypes. In each simulation, we randomly selected polymorphisms from the entire set of SNP markers in the MS-2000 populations. We only required that markers had a minor allele frequency (MAF) >0.05. It should be noted that we are modeling the randomly chosen marker as the causal polymorphism, whereas, as discussed above, in real GWA scans the marker is not always in perfect LD with the polymorphism.

**Figure 3:**
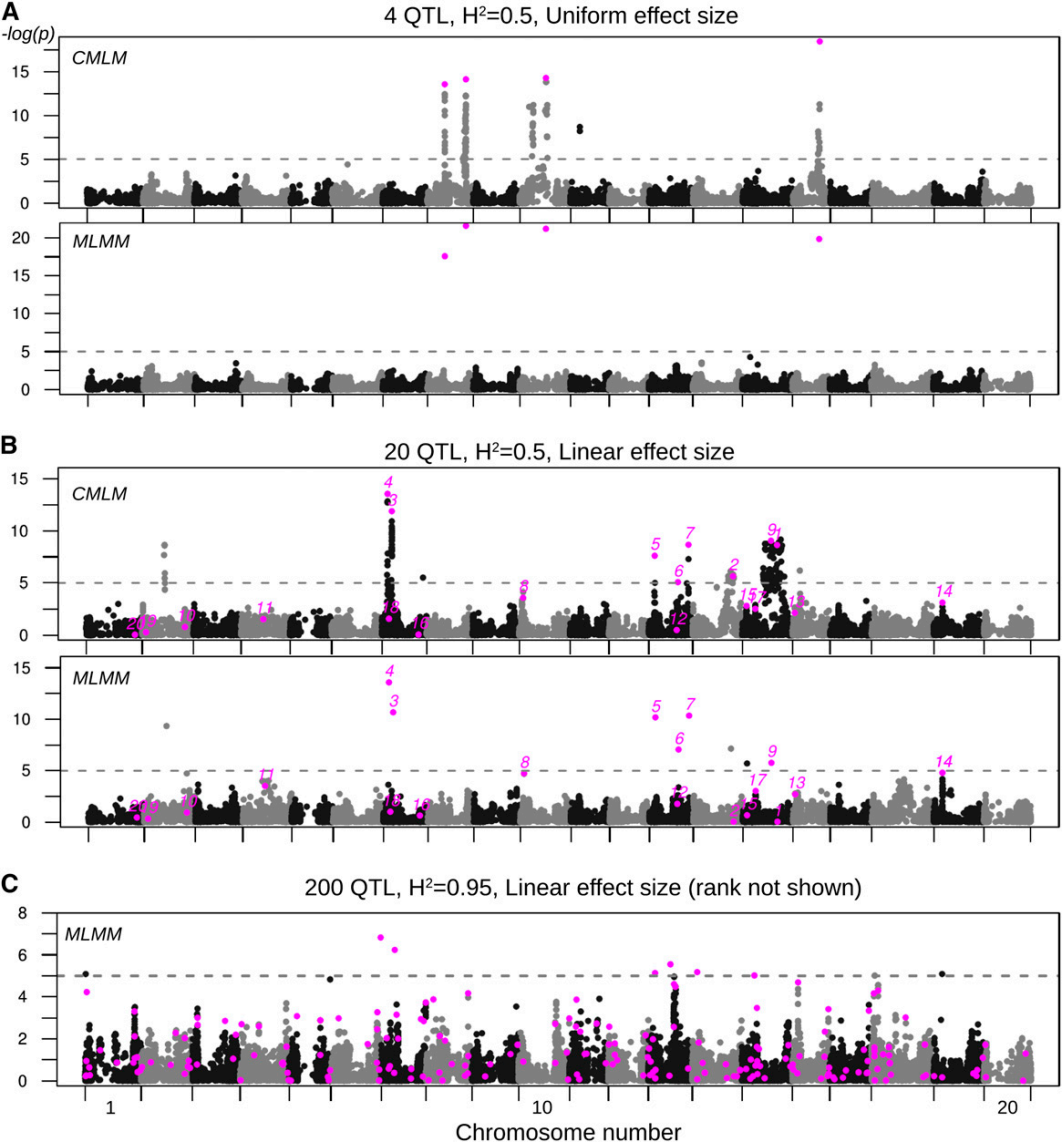
Significance scores of simulated genetic architectures in the MS-2000 soybean population using CMLM and MLMM methods. Each marker is plotted with its -log(p-val) on the y-axis and physical position is plotted on the x-axis. Chromosomes are indicated by alternating black and gray coloration and are plotted in order, 1 through 20. Magenta markers indicate the polymorphisms associated with a simulated effect. A significance threshold of p-value < 10^−5^ is indicated by a dotted line. (A) Four QTL with uniform effect sizes and a heritability of 0.5. (B) Twenty QTL sampled from linear effect sizes with a heritability of 0.5. Rank of the allelic effect is given above the marker, with “1” being the largest effect. (C) Two hundred QTL, a linear effect distribution, and a heritability of 0.95. Only MLMM method is shown.

For each simulation, we used both compressed mixed linear model (CMLM) and a multi-locus mixed model (MLMM) to identify significantly associated markers. CMLM approach corrects for genotypic background by incorporating pairwise kinship information and major principal components derived from a principal component analysis of the SNP information (Zhang *et al.* 2010). In addition to correcting for genetic background, the MLMM approach also includes markers discovered in initial cycles of the algorithm as co-factors in further iterations (Segura *et al.* 2012). We have found these approaches to be complementary in that MLMM effectively hides the LD between causal variants and distant markers; false positives in Figure 3 are effectively removed. Yet, to do this, the MLMM often must arbitrarily choose one marker from a broad peak of significance (Figure 3B). CMLM, however, retains this information; many markers have a significant association merely because they are in high LD with the causal variant. This information is useful when the LD is a result of physical linkage. For example, the variants with the top two strongest effects (“1” and “2”) in Figure 3B are not identified because others variants in linkage are selected by MLMM and included as covariates. It is clearer from the CMLM analysis that these loci have extensive LD. In this regard, rigorous evaluation of the success of an algorithm is difficult: MLMM can identify a neighboring marker instead of the causal polymorphism (“1” and “2” in Figure 3B), whereas CMLM will identify numerous markers around the causal polymorphism, complicating an assessment of false discovery rate. Any causal variant that was not significant at the given threshold in the CMLM model was considered a false negative (Table 1). Any marker that was not a causal variant but was deemed significant by both MLMM and CMLM was considered a false positive (Table 1). This evaluation strategy reflects the use of both statistical significance and visual analysis to determine true associations in real GWA scans.

Under the uniform effects distribution, each effect is simulated as the reciprocal of the number of QTL. Likewise, linear effects are, on average, smaller for traits under the control of a larger number of QTL. As expected, heritability plays a large role in detection success; in every condition, the statistical power declines with reduced heritability (Table 1). This is particularly important when variation in a trait is effected by a moderate number of variants. For linear effect distributions, the variants with the top quartile of effect sizes were, as expected, detected at much higher frequencies than the collection of all variants.

**Figure 4:**
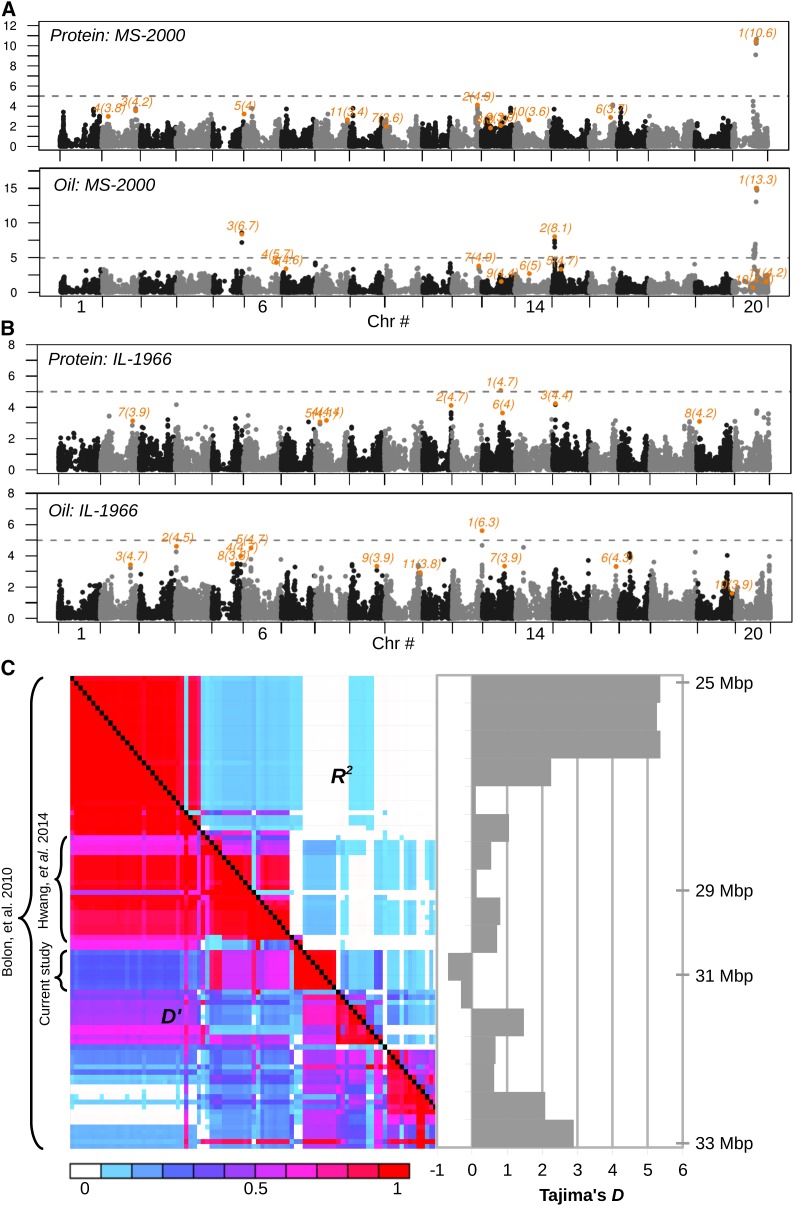
Percent protein and oil GWA scan. For population MS-2000 (A) and IL-1966 (B), each marker is plotted with its -log(p-val), as assessed using the CMLM method, on the y-axis and its physical position is plotted on the x-axis. Orange color indicates markers also identified by the MLMM method; their discovery order and -log(p-val) are also indicated in adjacent orange font. Chromosomes are indicated by alternating black and gray and are plotted in order, 1 through 20. A significance threshold of p-value < 10^−5^ is indicated by a dotted line. (C) Using MS-2000 population, LD plot for the region around the protein/oil QTL identified in this study and others. The total physical distance shown is ∼8 MB. R^2^ and D′’ measures of LD are given above and below the diagonal, respectively; both values range from 0 to 1. Brackets indicate the physical range previously found to associate with protein and oil content. The bar graph to the right of the LD plot is scaled approximately to the physical position along the LD plot, as indicated, and plots the Tajima’s D metric for sliding windows 20 markers wide with an overlap of 10 markers. Both monomorphic and polymorphic markers were included in the Tajima’s D calculation, whereas only polymorphic (MAF > 0.05) sites are shown in the LD plot.

The number of false positives does not fluctuate substantially across scenarios, although it is slightly higher under 20 QTL conditions. At an average frequency of ∼10^−4^ (ignoring the 2.3E−3 value, see below), one would expect one false positive per 10,000 markers tested at a threshold of p-value < 10^−5^. This value represents a ceiling on the false-positive frequency, because LD is still inflating our estimate. The outlying false-positive rate associated with the “20-QTL, uniform distribution, high-heritability” scenario appears to be the result of QTL being selected from a very large linkage block in two of the simulations (not shown). In our experience, visual selection of peaks using aggregate Manhattan plots (Figure 4) reduces the effective false-positive frequency to <1 per 30,000 markers.

As expected, in many tested scenarios the false-negative frequency declines with a reduced *p*-value threshold (Table 1). The compensatory increase in false-positive frequencies is often relatively small. Generally, increasing the threshold from 10^−5^ to 10^−4^ would add approximately three true positives per four false positive. For some studies or breeding objectives, this trade-off may be beneficial.

### Refining the protein and oil QTL using GWA scans

Applying insights from the simulation studies, we attempted to map QTL underlying protein and oil using GWA scans within both MS-2000 and IL-1966 populations discussed above, as well as two additional populations, IL-1964 and MS-1996 (Table S1). For the MS-2000 population, with mostly MG V accessions, we found a striking association between protein content and the 1-Mbp genomic region between 30,930,931 and 31,972,955 bp on chromosome (chr-) 20, with the greatest effect associated with the SNP marker at position 31,972,955 (Figure 4A). As discussed below, this marker is covered by the confirmed protein QTL region. Interestingly, the association on chr-20 is entirely absent when limiting the analysis to MG-III-IV lines grown in Illinois in 1966 (IL-1966) (Figure 4B), as well as MS-1996 and IL-1964 (Table 2).

Using family-based mapping, Bolon *et al.* (2010) narrowed the major protein QTL to an ∼8.5-Mbp region on chr-20 (Figure 4C). A recent study indicated that the known chr-20 protein QTL is located in the large LD block under this interval (Figure 4C). This region is approximately 1 Mbp upstream of the region that we identified. Using phenotypic data released as part of that study as well as 66% of the genotypes used therein (the remainder were unavailable), we ran a GWA scan and, as with the MS-2000 population, found that SNP marker BARC1.01Gm20_31610452 has the strongest association with protein content (Table 2).

On further analysis, we noted that in the MS-2000 population, all markers in moderate LD with BARC1.01Gm20_31972955 had very low minor allele frequencies; within the IL-1966 population, many of these markers are monomorphic. Using all monomorphic and polymorphic markers in the MS-2000 germplasm, we conducted a diversity analysis across this region (Figure 4C). Generally, a negative value of Tajima’s *D* is an indicator of purifying selection, whereas positive values are indicators of population bottlenecks (or diversifying selection). Soybean is known to have undergone a severe population bottleneck. In addition, ascertainment bias associated with our genotyping platform will result in higher values. Thus, the negative Tajima’s *D* values observed for this region occur against a background of higher than expected Tajima’s *D* values. Supporting this result, in a recent study that compared a *Glycine soja* population with a *G. max* population, Chung *et al.* (2013) identified the 30.5-to 32.3-Mbp region on chr-20 as being under selection related to domestication. It is possible that the low diversity of this region, which makes it very sensitive to MAF thresholds, caused it to be missed in previously published GWA scans (Huang *et al.* 2010).

GWA algorithms to control for population structure are less effective when the trait undergoing study has been under distinct modes of selection in distinct environments; flowering time is a classic example that has been notoriously difficult to map using population-based methods (Larsson *et al.* 2013). Such selection reduces the ability of gross genetic relatedness to predict the polygenic background effect (see Introduction). Because it is negatively correlated with oil and yield, selective pressure on protein content has probably been uniformly selected against, but there may have been conditions or environments in which protein content was favored (*e.g.*, for culinary reasons). By including a more accurate model of the background genetic effects of a trait in additional GWA scan iterations, the MLMM algorithm begins to address such issues (Segura *et al.* 2012), and generally the corroboration between CMLM and MLMM algorithms for this trait (Figure 4, A and B) suggests that selection is not confounding.

**Figure 5:**
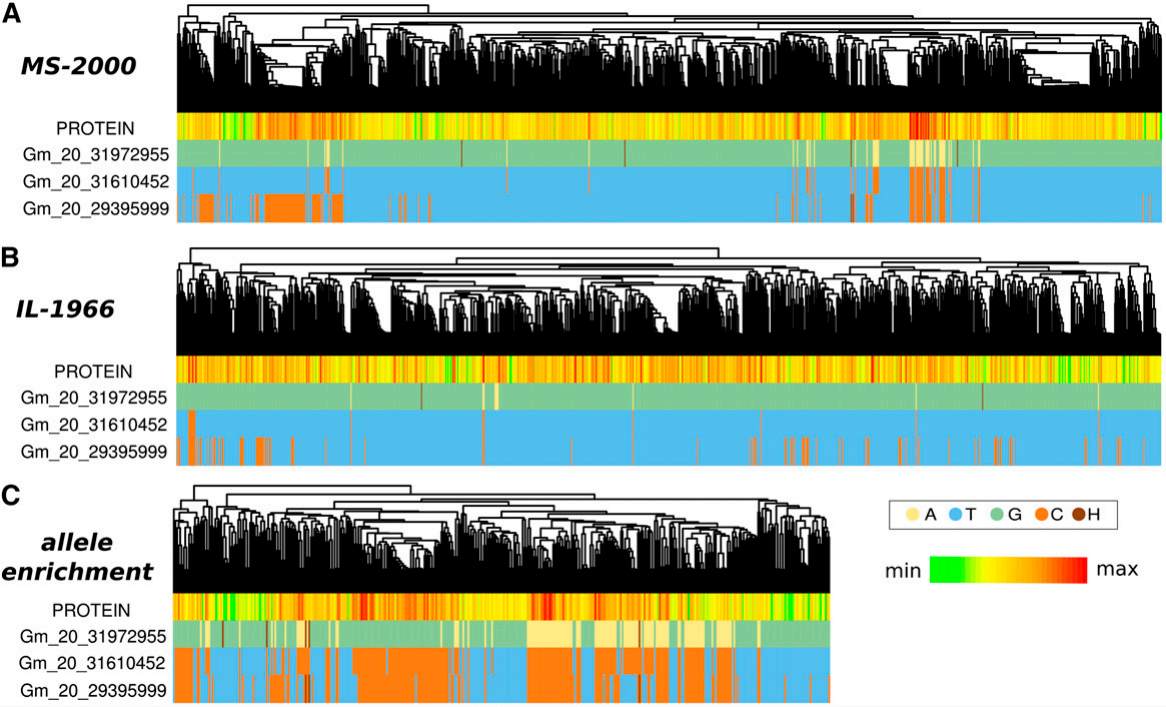
Allele distribution as it relates to population structure and protein levels. Genotypes were clustered based on genetic distance across all markers (not just those depicted here). Each genotype used in the study represents a leaf in the dendrogram at the top of each panel. Percent protein is represented as a heat map, with red being the highest value within a population and green being the lowest. For a given marker, a genotype is color-coded according to the nucleotide for which it is homozygous; heterozygotes are shown as brown. Marker names are abbreviated to exclude "BARC1.01." (A) MS-2000 population. (B) IL-1966 population. (C) Population created by enriching BARC1.01Gm20_31610452-C to a frequency of 0.5, regardless of the environment in which a line was phenotype.

For both MS-2000 and IL-1966 populations, we closely inspected the distribution of alleles residing at BARC1.01Gm 20_31972955, at BARC1.01Gm 20_31610452, which had a comparably low *p*-value, and also at BARC1.01Gm20_29395999, which is a marker that was identified previously (Hwang *et al.* 2014). The BARC1.01Gm 20_31972955-A allele clearly correlates with protein levels (Figure 5). Yet, with rare exceptions in the IL-1966 population, BARC1.01Gm 20_31972955-A is in complete LD with the BARC1.01Gm20_31610452-C allele. These exceptions, although few, suggest that BARC1.01Gm20_31610452 is more closely linked to the causative allele. Interestingly, the left branch of the MS-2000 population, although absent of both BARC1.01Gm 20_31972955-A and BARC1.01Gm20_31610452-C, has consistently higher than average protein levels (Figure 5A). This branch represents a fairly large group of genetically similar individuals; therefore, although the BARC1.01Gm20_29395999 allele appears to correlate broadly with protein levels, fine-scale analysis of more divergent individuals shows a more striking relationship between BARC1.01Gm20_31610452 and a line’s protein level. This relationship is reflected in the much lower *p*-value for BARC1.01Gm20_31610452 observed across multiple populations and algorithms (Table 2 and Figure 4).

In an attempt to further refine the interval identified above, we mined all genotyped accessions for which protein data were available, regardless of maturity group or environment. One-hundred ninety-two genotypes with the high protein marker, BARC1.01Gm20_31610452-C, were retained; an equal number of lines containing the major allele were randomly selected from the entire set. This enrichment yielded numerous examples of lines in which the linkage between BARC1.01Gm20_31610452-C and BARC1.01Gm 20_31972955-A is broken. There is a very distinct subpopulation that has BARC1.01Gm20_31610452-C, lacks BARC1.01Gm 20_31972955-A, and yet retains high protein levels (Figure 5C). Although BARC1.01Gm20_31610452-C is in high LD with BARC1.01Gm20_29395999-C, there are multiple examples where this linkage is broken; in those cases, protein levels still appear to be retained when BARC1.01Gm20_31610452-C is present (Figure 5C). Based on these results, BARC1.01Gm20_31610452 is the most precise marker for protein content yet to be identified.

As described above, oil and protein are highly correlated. It is still unknown if two QTL on chr-20 have close physical linkage or if a single QTL controls both phenotypes. In the oil GWA scan, the markers with the lowest significant p-values reside in the 31150270–31640038 bp linkage block, which supports the hypothesis that the same causal variant is controlling the strong negative correlation between traits. Yet, unlike the protein GWA scan, there appears to be multiple significant oil QTL. In addition to the chr-20 QTL, we also identified markers on chr-15, chr-5, and chr-6 in the MS-2000 population (Figure 4A). These same markers (or adjacent markers) also appear in two separate populations phenotyped in different environments (Table 2). The additional markers, although not as striking in effect as the chr-20 marker, may be a means to begin uncoupling protein and oil levels in soybean through the inclusion of positive effect oil markers in the presence of the high-protein chr-20 QTL. These markers overlap known QTL as reported in SoyBase (www.soybase.org, and references therein), specifically: BARC1.01Gm15_3919945 overlaps QTL Seed_Oil_32-1; BARC1.01Gm05_38495217 overlaps QTL Seed_Oil_30-1; and BARC1.01Gm6_42907701 overlaps QTL Seed_Oil_23_1 (QTL names are based on SoyBase indexing). It should be noted that 178 seed oil QTL are defined in SoyBase (May 22, 2014), and their summed intervals cover approximately one-eighth of the entire genome. Thus, for some traits, the coincidence between GWA scans and prior mapping results, although validating, are not definitive.

### Genes underlying protein quality

**Figure 6:**
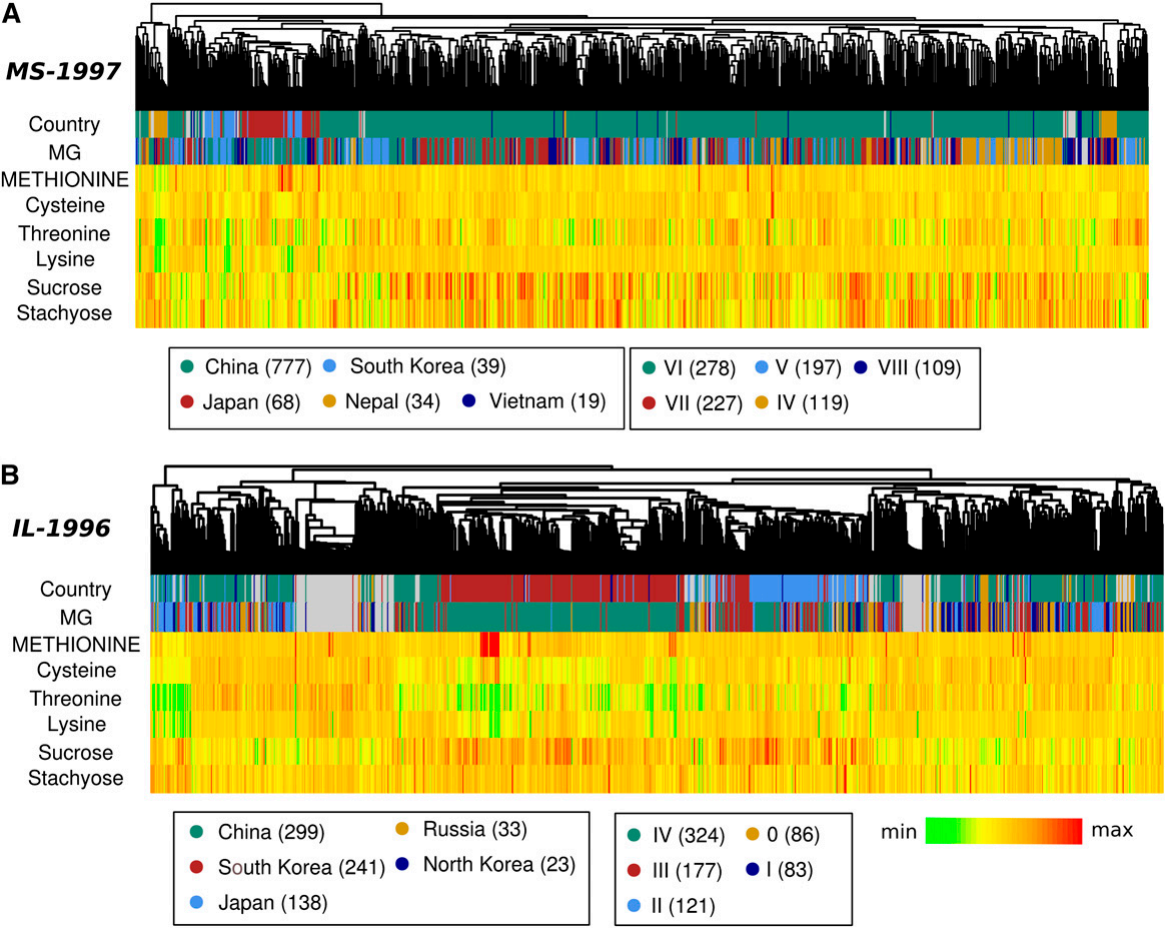
Attributes of populations used in GWA scans for seed quality traits. Genotypes were clustered based on genetic distance. Each genotype used in the study represents a leaf in the dendrogram at the top of each panel. Country of origin and maturity group (“MG”) are color-coded. Traits are represented as a heat map, with red being the highest value within a population and green being the lowest. Values in parentheses indicate the number of lines within a given category. (A) MS-1997 population. (B) IL-1996 population. Note that color-coding can be different for the same category in different populations.

As described in the Introduction, the value of protein meal could be dramatically improved by increasing its essential amino acid content, particularly with regard to methionine. Using a distinct set of germplasm that has been phenotyped for assorted sugars and amino acids (Table S1 and Figure 6), we investigated the genetic architecture underlying protein quality traits in soybean.

Both populations support the clustering of South Korean and Japanese germplasm relative to the more diverse Chinese germplasm (Figure 6), as seen with protein–oil populations above (Figure 2). Likewise, maturity group and genetic relatedness have a much weaker but still discernible relationship. For the IL-1996 population, Cys, Thr, and Lys traits have a mild correlation with one another. Additionally, these traits show a mild to weak negative correlation with Met.

Using GWA scans as above, we identified QTL that appear to be affecting protein quality. There is a strong association between Met levels and three separate loci within the IL-1996 population: BARC1.01Gm11_1657825; BARC1.01Gm20_694345; and BARC1.01Gm3_3936105 (Table 3 and Figure S1). All three of these markers fall below MAF thresholds in the MS-1997 population (Figure S3). Excluding these markers, the marker with the lowest *p*-value in IL-1996, BARC1.01Gm01_52253980, is adjacent to marker BARC1.01Gm01_52263952, which has the lowest *p*-value in 1997-MS. Within the IL-1996 population, there is a group of closely related individuals that share very high Met levels (Figure 6), and we were concerned that even after accounting for the effect of population structure, this subpopulation was confounding GWA results. Removing all but one representative from this subpopulation and rerunning the analysis gave comparable results, although the *p*-values were higher for each marker, and BARC1.01Gm20_694345 was removed because of low MAF (data not shown). Multiple closely spaced markers on chr-8 associate with Cys, Lys, and Thr, and these relationships hold across populations (Table 3 and Figure S1). Likewise, BARC1.01Gm01_52253980 and adjacent markers have pan-trait, pan-population significance (Table 3 and Figure S1). Thus, there is clearly genetic overlap between essential amino acid traits, as expected when compounds are synthesized by shared biochemical pathways, and it remains a challenge to understand the molecular details of this overlap. The identification of high-resolution Met-specific QTL (Table 3) holds promise for fine-mapping genes responsible for relative amino acid content.

The effect of maturity date on Met-Cys profiles has been observed in biparental mapping populations (see Introduction), and we see a minor effect of maturity group on amino acid composition. In part, this is due to a weak relationship between maturity group and population structure, particularly in IL-1996 (Figure 6), and should be accounted for in both MLMM and CMLM approaches. Results are comparable when limiting our GWA scans to only those genotypes within the most abundant maturity group of a given population—IV for IL-1996 and VI for MS-1997—particularly with regard to markers that are identified across populations (not shown). Those markers that are missed when limiting to specific maturity groups are generally missed because they fall below the specified MAF threshold.

Sucrose and stachyose exhibit low heritability relative to other seed quality traits (Table 3) as observed in biparental mapping studies (Wang *et al.* 2014), although estimates can vary dramatically (Kim *et al.* 2006). Although large-effect QTL have been identified in such crosses (Kim *et al.* 2005), GWA scans were unable to uncover such QTL (Table 3 and Figure S2). It can be difficult to identify sucrose QTL across biparental crosses even when the same parent is used (Kim *et al.* 2005, 2006). Thus, sugar traits appear to be resistant to GWA scans, either because large-effect alleles are rare or because strong genotype-by-environment effects confound results.

Family-based mapping studies of Met and Cys content only identified two QTL with an additive effect >0.1. Markers for these QTL were located on chr-13 and chr-18 (Panthee *et al.* 2006a). We did not find a significant association between either trait and any marker on chr-13. Met has a weak association with a SNP on chr-18, and this overlaps the interval of the previously discovered marker, Satt564. It is common for family-based studies to have distinct results from GWA scans. Population-based studies, such as GWA, will miss rare alleles, whereas these alleles will be enriched, at least theoretically, to 50% in family-based, bi-parental crosses. Alternatively, many alleles in a population that influence a trait may be missing in family-based studies and therefore cannot be identified. Nested-association mapping (NAM) populations in soybean will likely address some of these issues, although these populations will likely have reduced resolution and allelic diversity relative to GWA scans using large, heterogeneous panels, such as those described here.

## Conclusions

Modern soybean breeders are presented with a range of crop improvement techniques, such as marker-assisted selection (Xu and Crouch 2008), genomic selection (Heffner *et al.* 2009), and novel engineering strategies (Lusser *et al.* 2012), among others. The genetic architecture of the trait of interest heavily influences which breeding strategy will be most effective. Additionally, the more precisely the physical position of a gene can be determined, the more efficient the chosen strategy will be. Enabled by high-density marker development, GWA scans offer a genetic resolution limited only by the LD of the worldwide germplasm. In this study, GWA scans have allowed us to identify soybean seed composition markers with <1 Mbp resolution. In addition, we can differentiate those markers whose effects are shared between distinct germplasm pools. Taking these characteristics together, these markers should be of great utility across the diverse range of current breeding programs.

## Supplementary Material

Supporting Information
