# Supplementary material for: The Genetic Architecture of Seed Composition in Soybean Is Refined by Genome-Wide Association Scans Across Multiple Populations
Source: G3 (Bethesda). 2014 Sep 22;4(11):2283–94. doi: 10.1534/g3.114.013433 (PMC4232554; doi:10.1534/g3.114.013433)
Supplement: Supporting Information [file supp_4_11_2283__index.html]

The Genetic Architecture of Seed Composition in Soybean Is Refined by Genome-Wide Association Scans Across Multiple Populations — Supporting Information 

# The Genetic Architecture of Seed Composition in Soybean Is Refined by Genome-Wide Association Scans Across Multiple Populations

## Supporting Information for Vaughn *et al.*, 2014

**Files in this Data Supplement:**

- Supporting Information - Figures S1-S7, Table S1, and File S1 (PDF, 2 MB)
- Figure S1 - GWA scan results for multiple essential amino acid levels in environment/population datasets MS-1997 and IL-1996. (PDF, 1 MB)
- Figure S2 - GWA scan results for sucrose and stachyose levels in environment/population datasets MS-1997 and IL-1996. (PDF, 1 MB)
- Figure S3 - Allele distribution as it relates to population structure and methionine levels. (PDF, 1 MB)
- Figure S4 - PCA plots based on genetic relatedness in the IL-1966 population used for protein and oil GWAS. (PDF, 1 MB)
- Figure S5 - PCA plots based on genetic relatedness in the MS-2000 population used for protein and oil GWAS. (PDF, 1 MB)
- Figure S6 - PCA plots based on genetic relatedness in the IL-1996 population used for amino acid GWAS. (PDF, 1 MB)
- Figure S7 - PCA plots based on genetic relatedness in the MS-1997 population used for amino acid GWAS. (PDF, 1 MB)
- Table S1 - Phenotyping locations and dates for all populations reported in this study. (PDF, 1 MB)
- File S1 - Excel file containing averaged phenotypic values used in this study. (.xls, 583 KB)
