# Supplementary material for: The Genetic Architecture of Seed Composition in Soybean Is Refined by Genome-Wide Association Scans Across Multiple Populations
Source: G3 (Bethesda). 2014 Sep 22;4(11):2283–94. doi: 10.1534/g3.114.013433 (PMC4232554; doi:10.1534/g3.114.013433)
Supplement: Supporting Information [file supp_g3.114.013433_013433SI.pdf]

G3: Genes | Genomes | Genetics

THE GENETIC ARCHITECTURE OF SEED COMPOSITION IN SOYBEAN IS REFINED BY GENOME-WIDE ASSOCIATION SCANS ACROSS MULTIPLE POPULATIONS

Justin N. Vaughn[*], Randall L. Nelson[§], Qijian Song[†], Perry B. Cregan[†], and Zenglu Li[*]

[*]Center for Applied Genetic Technologies and Department of Crop and Soil Sciences, University of Georgia, Athens, GA 30602

[§]Soybean Maize Germplasm, Pathology, and Genetics Research Unit, US DA, Agricultural Research Service, and Department of Crop Sciences University of Illinois, Urbana, IL 61801

[†]Soybean Genomics and Improvement Laboratory, U.S. Department of Agriculture, Agricultural Research Service, Beltsville, MD 20705

Corresponding author:
Zenglu Li
Center for Applied Genetic Technologies
The University of Georgia
111 Riverbend Road
Athens, GA 30602-6810
phone: 706/542-9805
email: zli@uga.edu

DOI: 10.1534/g3.114.013433

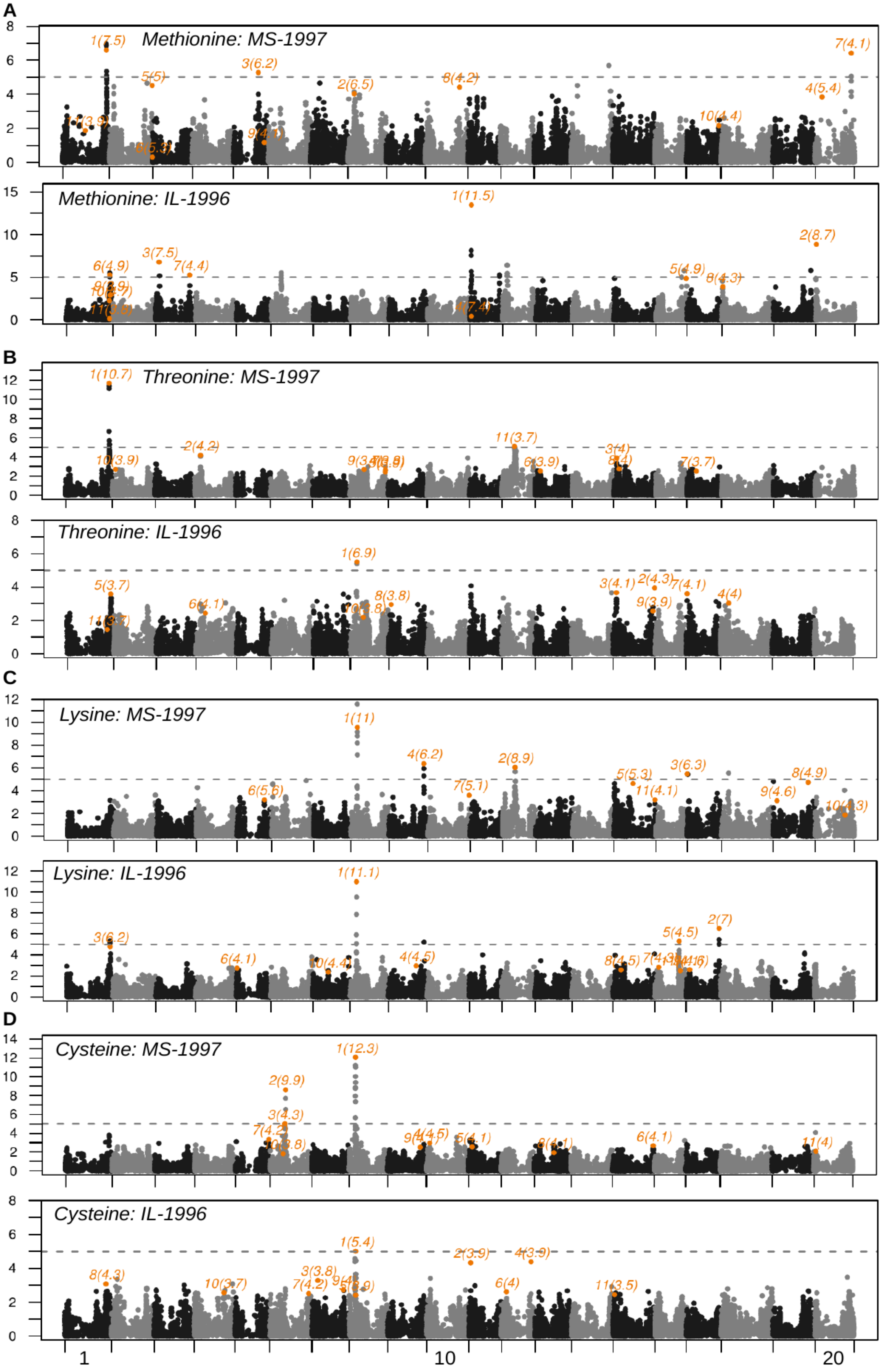
A
Methionine: MS-1997
Methionine: IL-1996
B
Threonine: MS-1997
Threonine: IL-1996
C
Lysine: MS-1997
Lysine: IL-1996
D
Cysteine: MS-1997
Cysteine: IL-1996
1
10
20

2 SI

Figure S1 (previous page): GWA scan results for multiple essential amino acid levels in environment/population datasets MS-1997 and IL-1996. Each marker is plotted with its -log(*p*-val), as assessed using the CMLM method, on the y-axis and its physical position on the x-axis. Orange coloration indicates markers also identified by the MLMM method; their rank and -log(*p*-val) are also indicated in adjacent orange font. Chromosomes are indicated by alternating black and gray coloration and are plotted in order, 1 through 20. A significance threshold of p-val<10^{-5} is indicated by a dotted line; though arbitrarily chosen, it allows comparison across all GWAS scans in this study. A) Methionine. B) Threonine. C) Lysine. D) Cysteine.

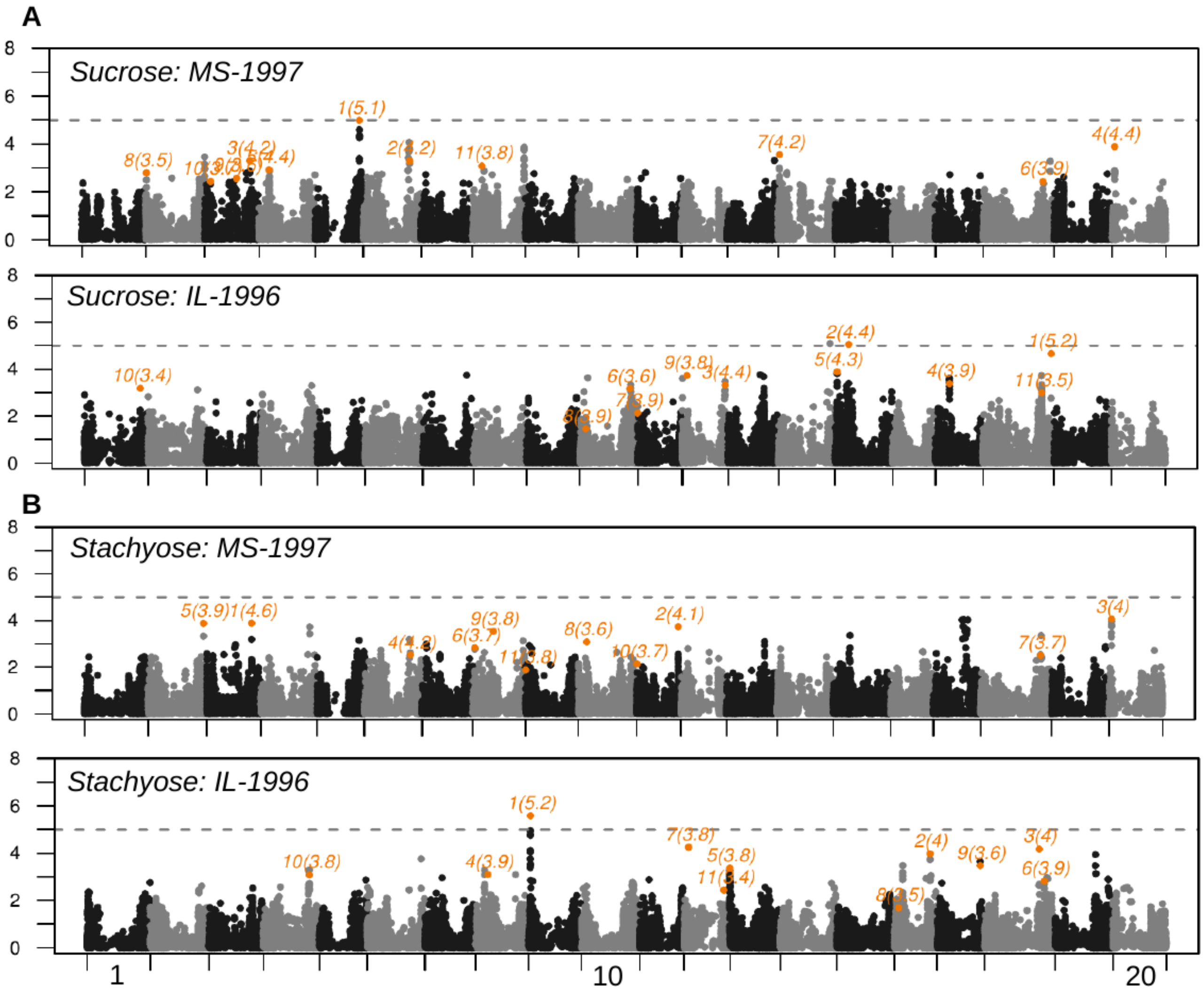

Figure S2: GWA scan results for sucrose and stachyose levels in environment/population datasets MS-1997 and IL-1996. Each marker is plotted with its -log(*p*-val), as assessed using the CMLM method, on the y-axis and its physical position on the x-axis. Orange coloration indicates markers also identified by the MLMM method; their rank and -log(*p*-val) are also indicated in adjacent orange font. Chromosomes are indicated by alternating black and gray coloration and are plotted in order, 1 through 20. A significance threshold of p-val<10^{-5} is indicated by a dotted line; though arbitrarily chosen, it allows comparison across all GWAS scans in this study. A) Sucrose. B) Stachyose.

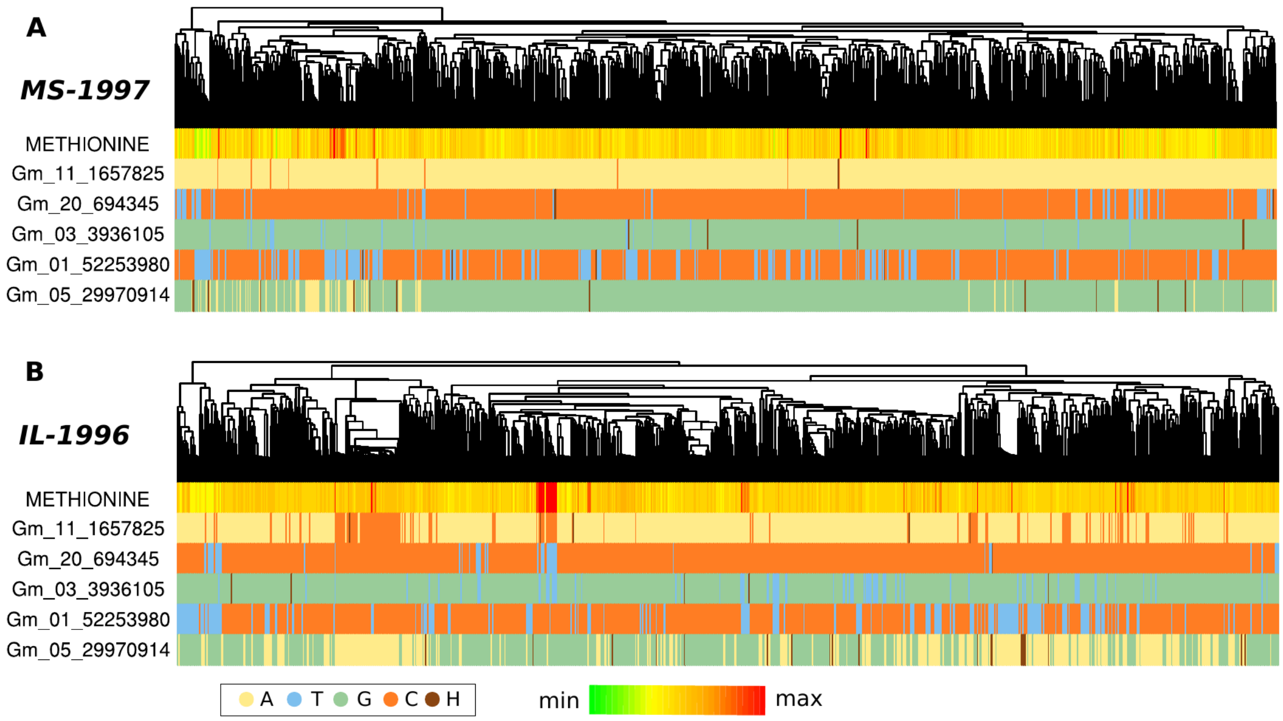

Figure S3: Allele distribution as it relates to population structure and methionine levels. Genotypes were clustered based on genetic distance across all markers (not just those depicted here). Each genotype used in the study represents a leaf in the dendrogram at the top of each panel. Methionine level is represented as a heat map with red being the highest value within a population and green being the lowest. For a given marker, a genotype is color coded according to the nucleotide for which it is homozygous; heterozygotes are shown as brown. A) MS-1997 population. B) IL-1996 population.

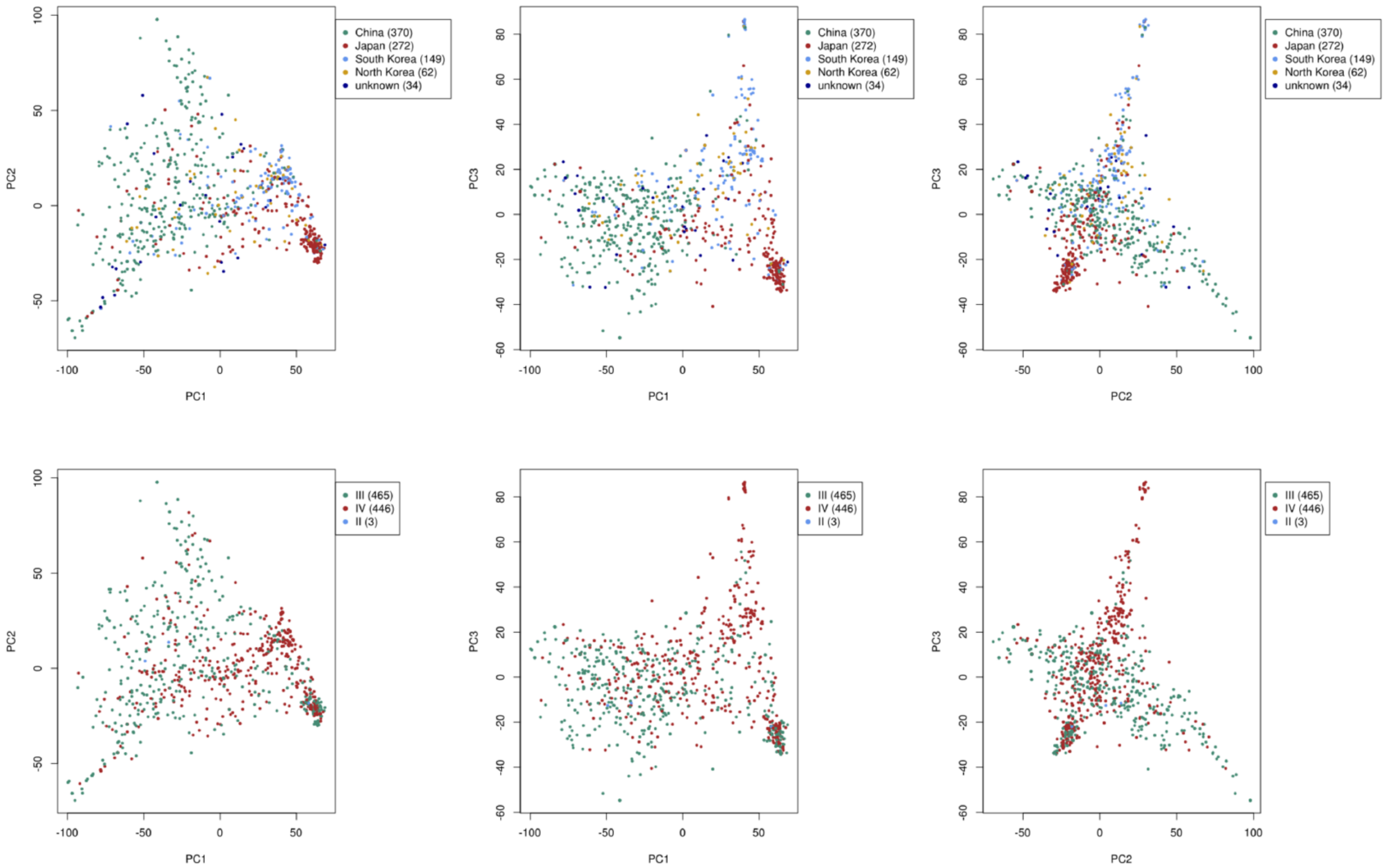

Figure S4: Principal component analysis (PCA) plots based on SNP information in the IL-1966 population used for protein and oil GWAS. Country of origin is indicated in the top row. Maturity group is indicated in the bottom row.

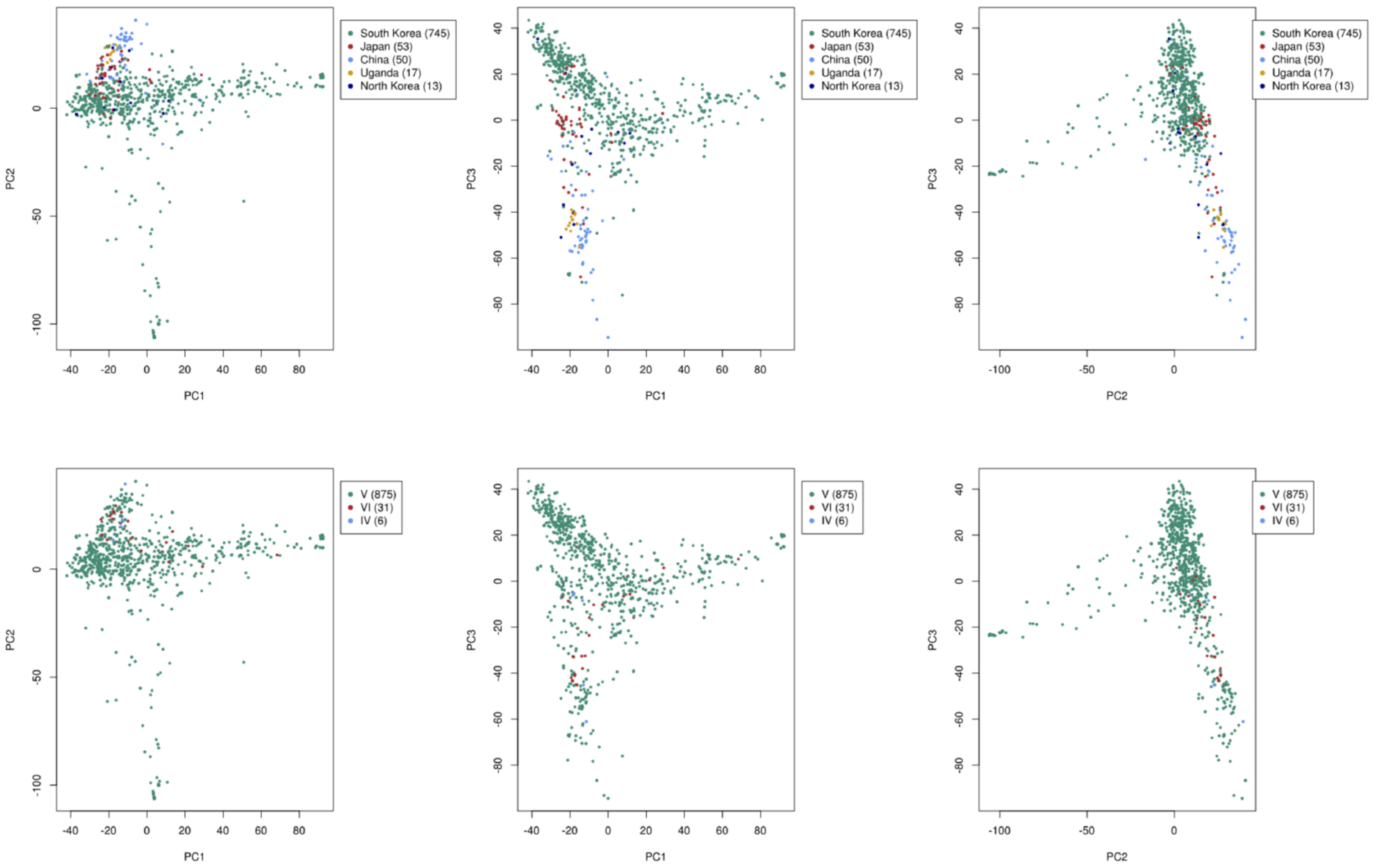

Figure S5: PCA plots based on SNP information in the MS-2000 population used for protein and oil GWAS. Country of origin is indicated in the top row. Maturity group is indicated in the bottom row.

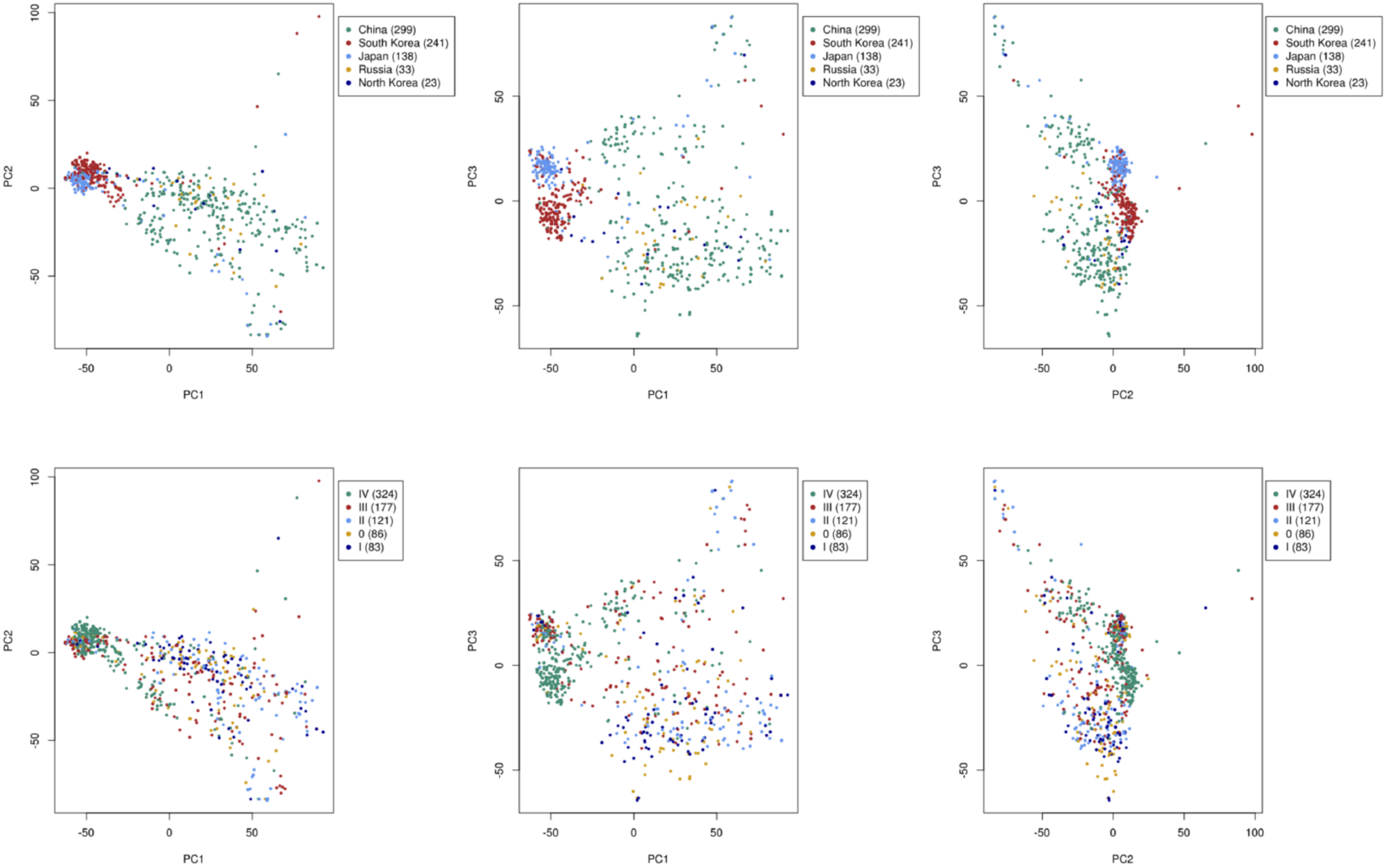

Figure S6: PCA plots based on SNP information in the IL-1996 population used for amino acid GWAS. Country of origin is indicated in the top row. Maturity group is indicated in the bottom row.

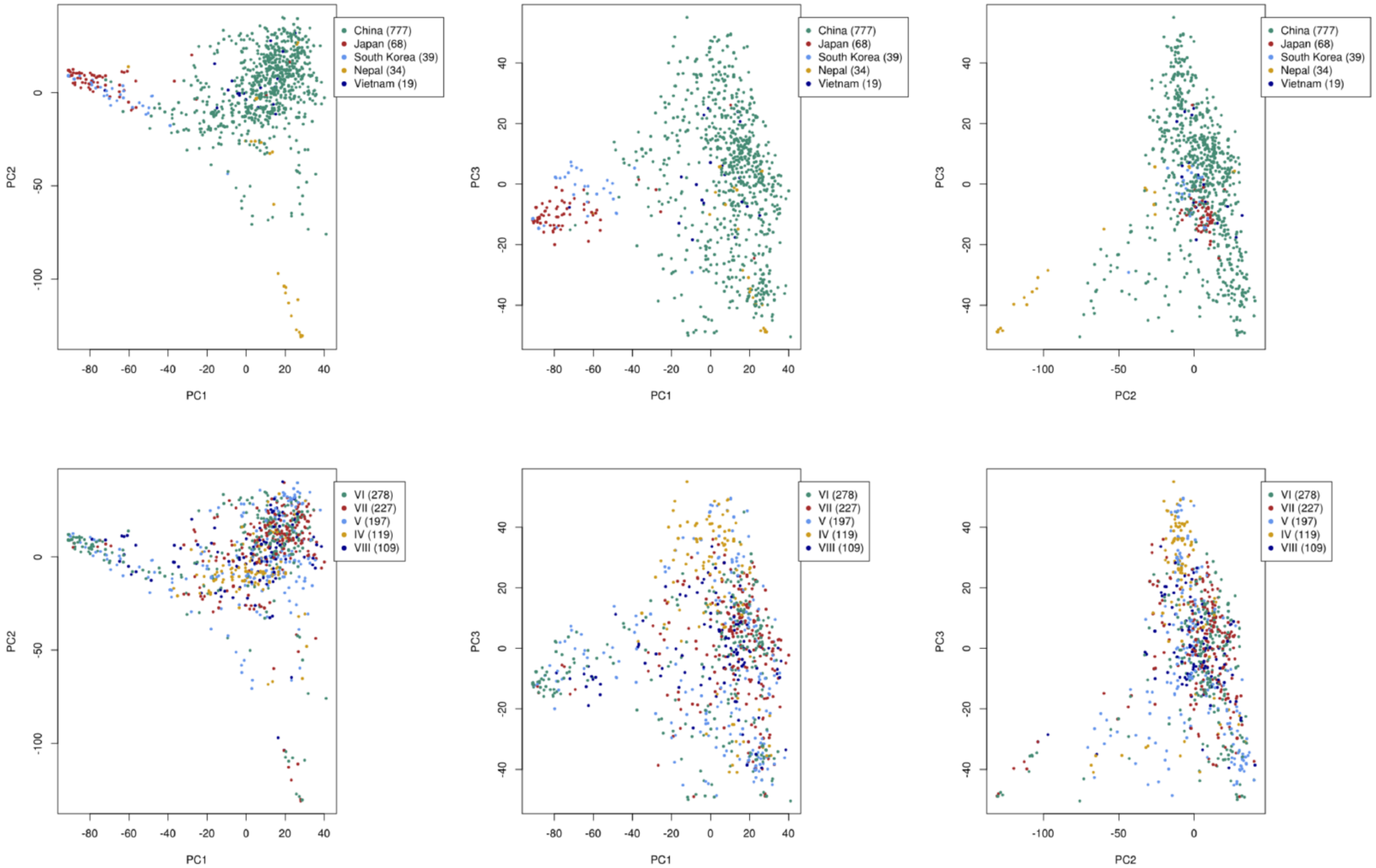

Figure S7: PCA plots based on SNP information in the MS-1997 population used for amino acid GWAS. Country of origin is indicated in the top row. Maturity group is indicated in the bottom row.

Table S1. Phenotyping locations and dates for all populations reported in this study. Populations within a particular "traits analyzed" category do not overlap with regard to genotype.

Traits analyzed	Population name	Location	Replication structure	Number of genotypes	Number of SNPs used
Protein and oil	IL-1964	Urbana, IL	1964(2), average	619	32,396
	IL-1966	Urbana, IL	1965(1) & 1966(1), average	977	33,265
	MS-1996	Stoneville, MS	1996(1) and 1997(1), average	728	32,328
	MS-2000	Stoneville, MS	1999(1) & 2001(1), average	934	28,622
Met, Cys, Lys, Thr, sucrose, and stachyose	IL-1996	Urbana, IL	1996(1)	900	34,479
	MS-1997	Stoneville, MS	1997(1)	978	32,112

[a] Value in parentheses is the number of replications phenotyped in that year

File S1

Averaged phenotypic values used in this study

Available for download as an Excel file at
http://www.g3journal.org/lookup/suppl/doi:10.1534/g3.114.013433/-/DC1
