# Supplementary material for: The Genetic Architecture of Seed Composition in Soybean Is Refined by Genome-Wide Association Scans Across Multiple Populations
Source: G3 (Bethesda). 2014 Sep 22;4(11):2283–94. doi: 10.1534/g3.114.013433 (PMC4232554; doi:10.1534/g3.114.013433)
Supplement: Supporting Information [file supp_g3.114.013433_FigureS2.pdf]

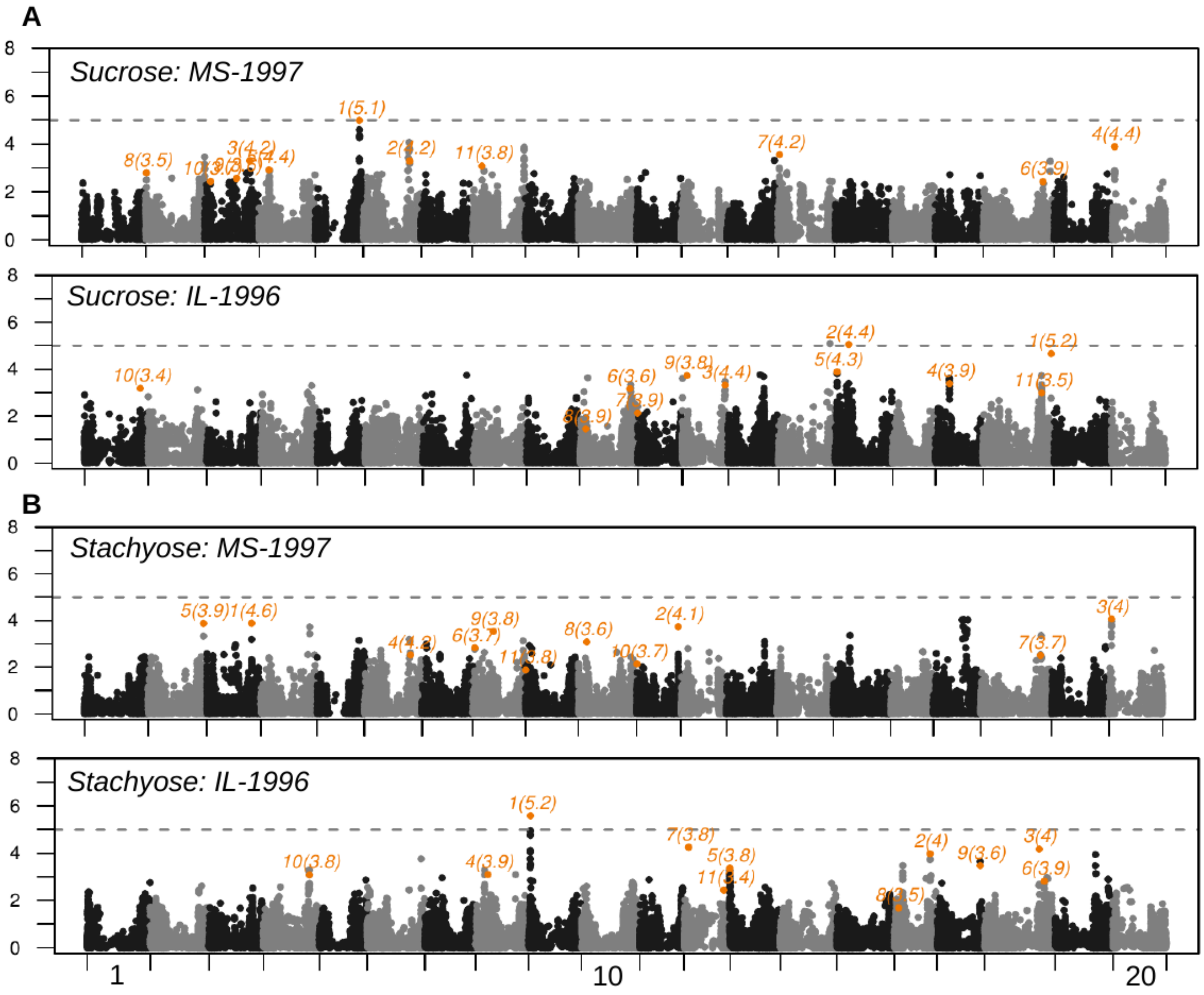

Figure S2: GWA scan results for sucrose and stachyose levels in environment/population datasets MS-1997 and IL-1996. Each marker is plotted with its -log(*p*-val), as assessed using the CMLM method, on the y-axis and its physical position on the x-axis. Orange coloration indicates markers also identified by the MLMM method; their rank and -log(*p*-val) are also indicated in adjacent orange font. Chromosomes are indicated by alternating black and gray coloration and are plotted in order, 1 through 20. A significance threshold of p-val<10^{-5} is indicated by a dotted line; though arbitrarily chosen, it allows comparison across all GWAS scans in this study. A) Sucrose. B) Stachyose.
