# Supplementary material for: The Genetic Architecture of Seed Composition in Soybean Is Refined by Genome-Wide Association Scans Across Multiple Populations
Source: G3 (Bethesda). 2014 Sep 22;4(11):2283–94. doi: 10.1534/g3.114.013433 (PMC4232554; doi:10.1534/g3.114.013433)
Supplement: Supporting Information [file supp_g3.114.013433_FigureS3.pdf]

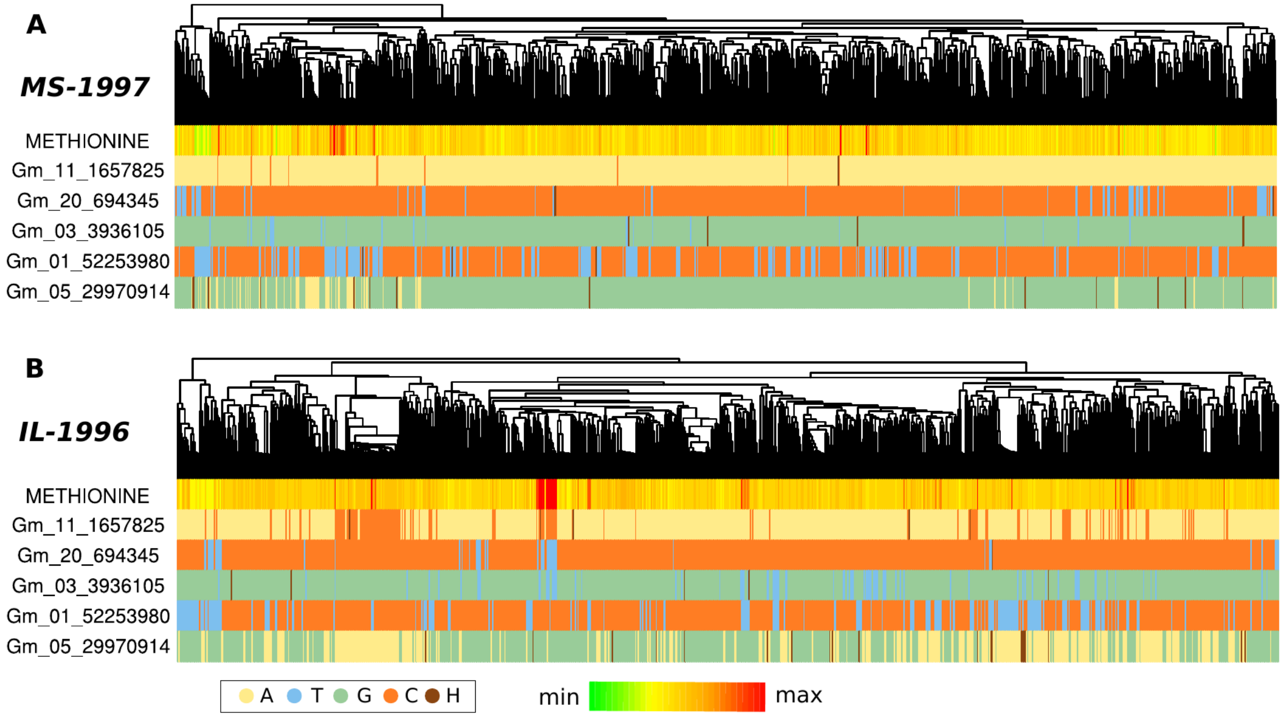

Figure S3: Allele distribution as it relates to population structure and methionine levels. Genotypes were clustered based on genetic distance across all markers (not just those depicted here). Each genotype used in the study represents a leaf in the dendrogram at the top of each panel. Methionine level is represented as a heat map with red being the highest value within a population and green being the lowest. For a given marker, a genotype is color coded according to the nucleotide for which it is homozygous; heterozygotes are shown as brown. A) MS-1997 population. B) IL-1996 population.
