# Supplementary material for: The Genetic Architecture of Seed Composition in Soybean Is Refined by Genome-Wide Association Scans Across Multiple Populations
Source: G3 (Bethesda). 2014 Sep 22;4(11):2283–94. doi: 10.1534/g3.114.013433 (PMC4232554; doi:10.1534/g3.114.013433)
Supplement: Supporting Information [file supp_g3.114.013433_FigureS5.pdf]

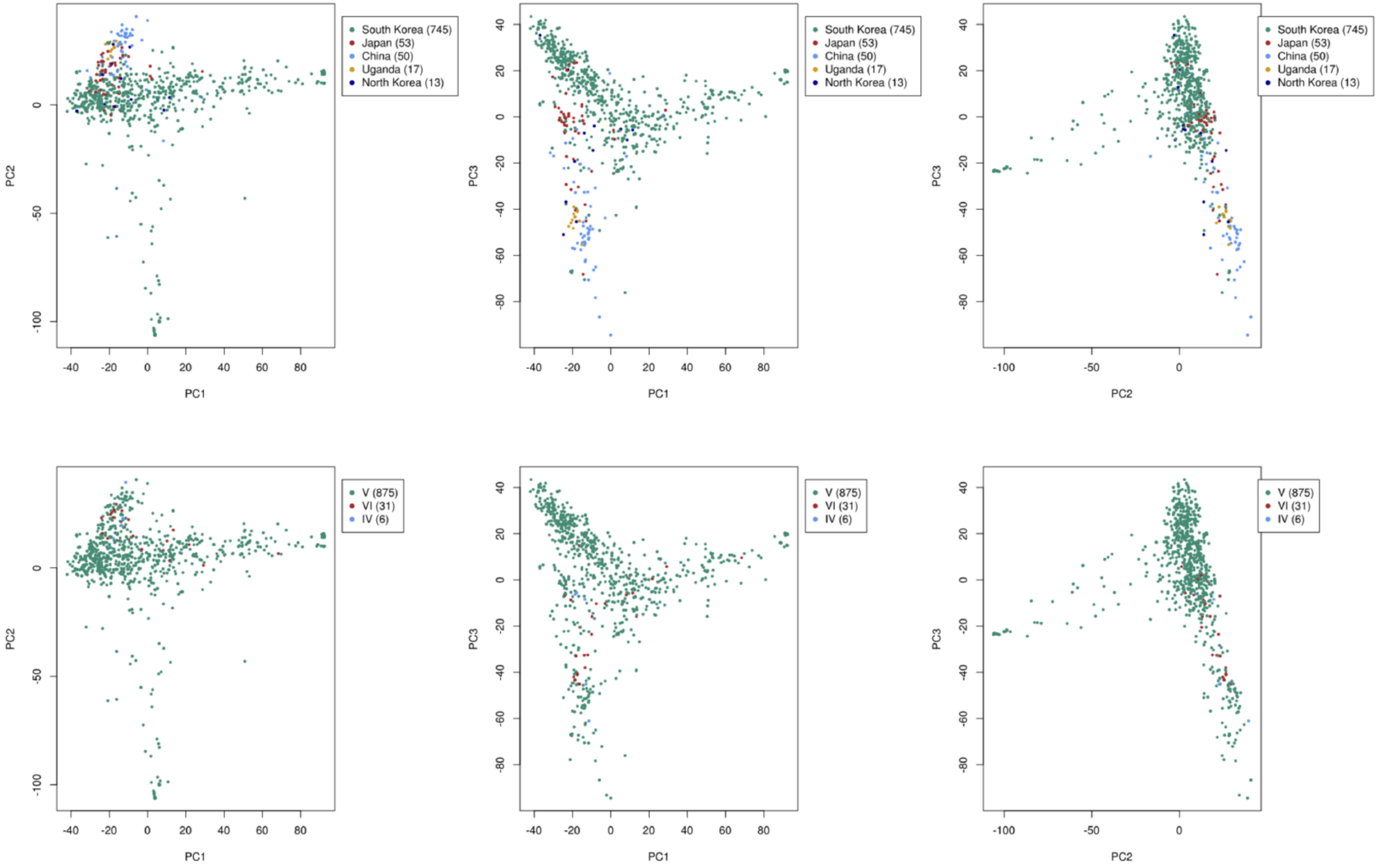

Figure S5: PCA plots based on SNP information in the MS-2000 population used for protein and oil GWAS. Country of origin is indicated in the top row. Maturity group is indicated in the bottom row.
