# Supplementary material for: The Genetic Architecture of Seed Composition in Soybean Is Refined by Genome-Wide Association Scans Across Multiple Populations
Source: G3 (Bethesda). 2014 Sep 22;4(11):2283–94. doi: 10.1534/g3.114.013433 (PMC4232554; doi:10.1534/g3.114.013433)
Supplement: Supporting Information [file supp_g3.114.013433_TableS1.pdf]

Table S1. Phenotyping locations and dates for all populations reported in this study. Populations within a particular "traits analyzed" category do not overlap with regard to genotype.

Traits analyzed	Population name	Location	Replication structure	Number of genotypes	Number of SNPs used
Protein and oil	IL-1964	Urbana, IL	1964(2), average	619	32,396
	IL-1966	Urbana, IL	1965(1) & 1966(1), average	977	33,265
	MS-1996	Stoneville, MS	1996(1) and 1997(1), average	728	32,328
	MS-2000	Stoneville, MS	1999(1) & 2001(1), average	934	28,622
Met, Cys, Lys, Thr, sucrose, and stachyose	IL-1996	Urbana, IL	1996(1)	900	34,479
	MS-1997	Stoneville, MS	1997(1)	978	32,112

[a] Value in parentheses is the number of replications phenotyped in that year
